# Supplementary material for: HMGN1 enhances CRISPR-directed dual-function A-to-G and C-to-G base editing
Source: Nat Commun. 2023 Apr 27;14:2430. doi: 10.1038/s41467-023-38193-2 (PMC10140177; doi:10.1038/s41467-023-38193-2)
Supplement: Supplementary file 1 — Supplementary Information [file 41467_2023_38193_MOESM1_ESM.pdf]

HMGN1 enhances CRISPR-directed dual-function A-to-G and C-to-G base editing

Yang et al.

Supplementary Information

Supplementary figures

Supplementary Fig. 1

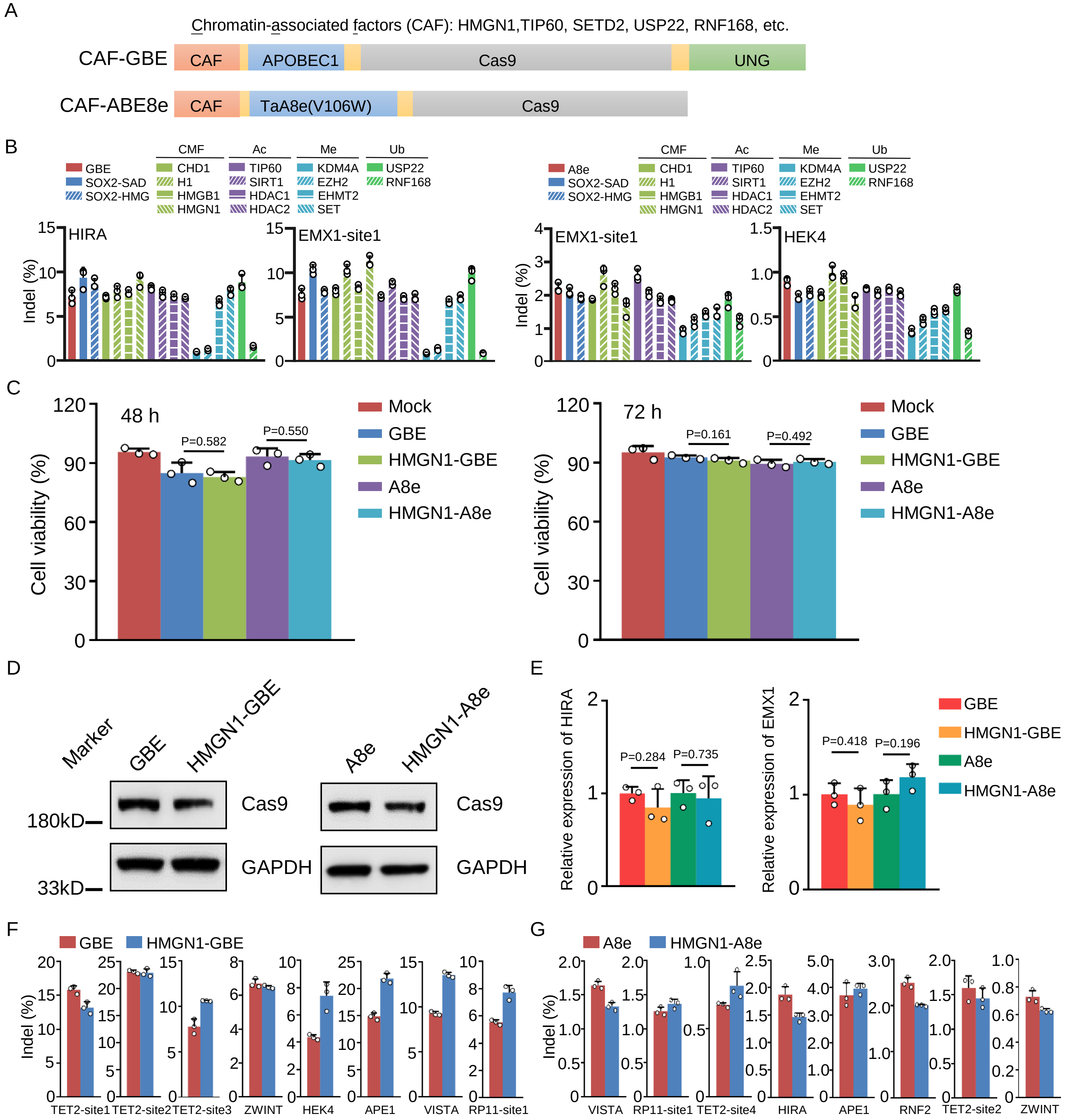

Supplementary figures

Supplementary Fig. 1

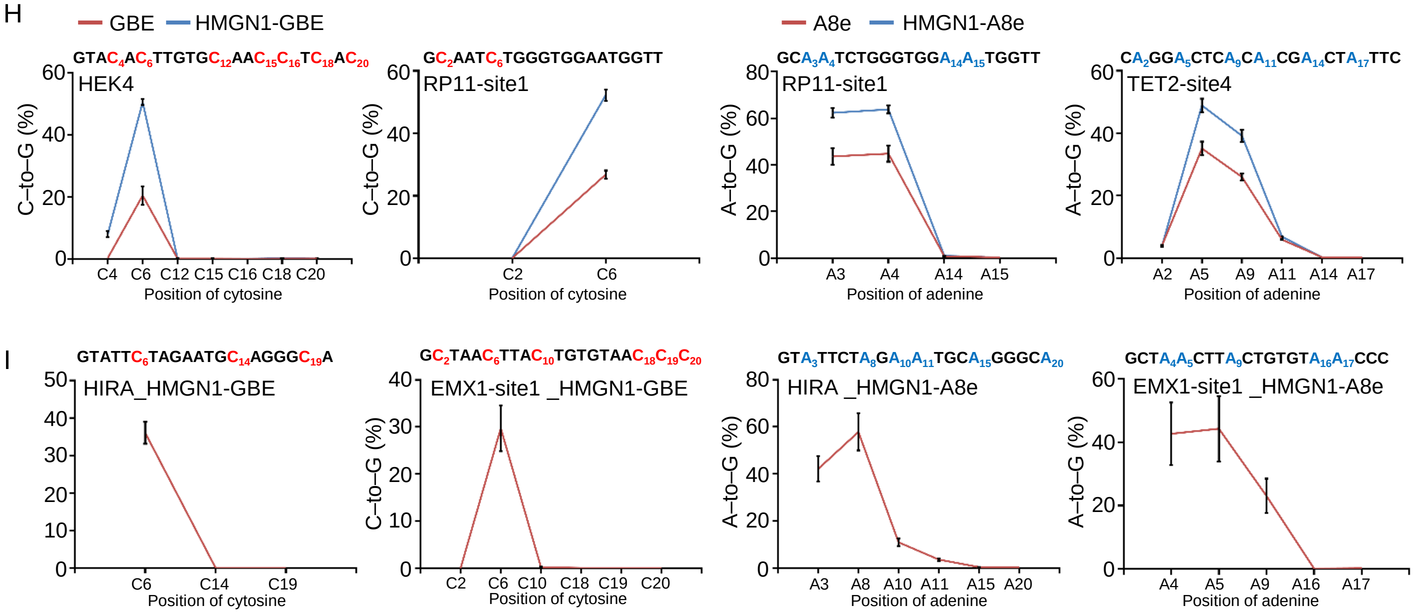

Figure S1. Further characterization of HMGN1-fused base editors and potentially adverse effects with HMGN1 fusion. (A) Schematic of base editor variant with fusion of chromatin associated factors. (B) Indel frequency across GBE (left) and ABE (right) variants fused with sixteen chromatin associated factors at HIRA and EMX1-site1 loci in HEK293T cells. (C) Comparison of cell viability between GBE and ABE variants at 48 h and 72 h. (D) Comparison of protein expression between GBE and ABE variants in HEK293T cells. (E) Comparison of relative mRNA level in HIRA and EMX1 between GBE and ABE variants. (F) Comparison of indel frequency between GBE and HMGN1-GBE across eight genomic loci in HEK293T cells. (G) Comparison of indel frequency between A8e and HMGN1-A8e across eight genomic loci in HEK293T cells. (H) Comparison of editing efficiency between GBE and ABE variants in HeLa cells. (I) Editing frequency of HMGN1-GBE and HMGN1-A8e at HIRA and EMX1-site1 in primary prostate carcinoma cells. Mean ± SEM (B, C, E-I) of all individual values of sets of n = 3 independent replicates are shown. All statistical analysis for samples were conducted using unpaired Student's t-test (two-tailed) in GraphPad Prism 8. Source data are provided as a Source Data file.

Supplementary figures

Supplementary Fig. 2

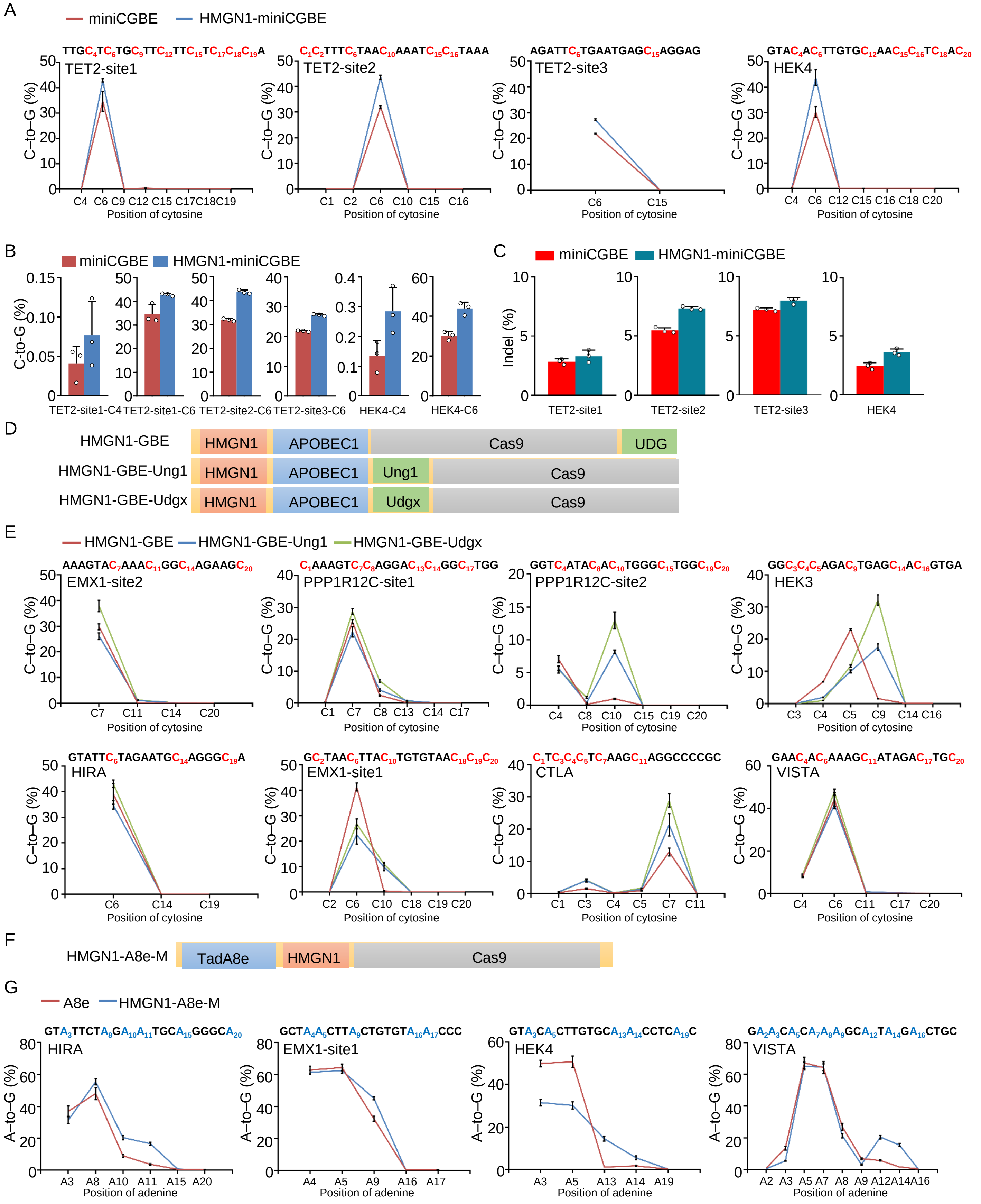

Supplementary figures

Figure S2. Verification and further optimization of C-to-G and A-to-G base editor with HMGN1. (A) Comparison of editing frequency between miniCGBE and HMGN1-miniCGBE at four genomic loci in HEK293T cells. (B) Comparison of editing frequency at indicated cytosines between miniCGBE and HMGN1-miniCGBE. (C) Comparison of indel frequency between miniCGBE and HMGN1-miniCGBE at four genomic loci in HEK293T cells. (D) Schematic of base editor variant with fusion of diverse glycosylases. (E) Comparison of editing frequency across HMGN1-GBE, HMGN1-GBE-Ung1, HMGN1-GBE-Udgx at eight genomic loci in HEK293T cells. (F) Schematic of HMGN1-A8e-M (HMGN1-A8e-Middle) variant. (G) Comparison of editing frequency between A8e and HMGN1-A8e-M at four genomic loci in HEK293T cells. Mean ± SEM (A-C, E, G) of all individual values of sets of n = 3 independent replicates are shown. Source data are provided as a Source Data file.

Supplementary figures

Supplementary Fig. 3

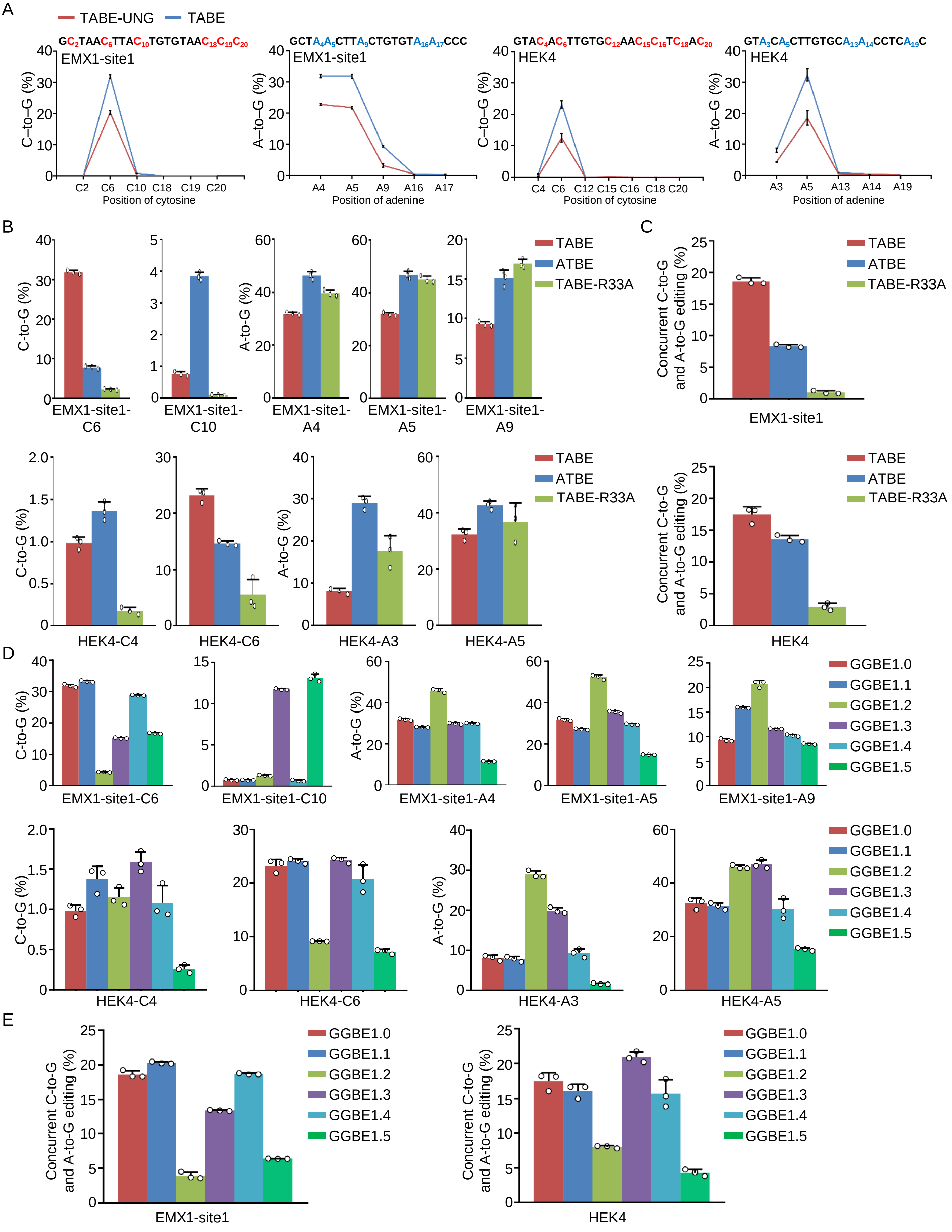

Supplementary figures

Supplementary Fig. 3

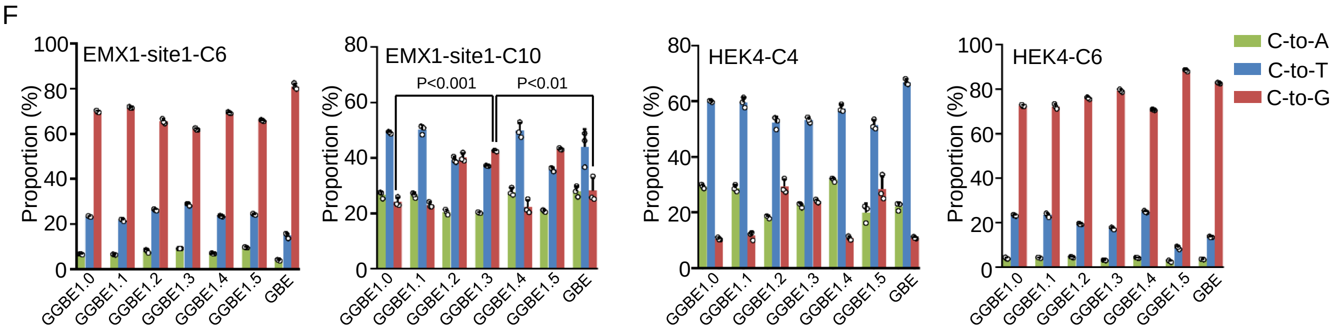

Figure S3. Editing efficiency and purity across GGBE and its variants. (A) Comparison of editing frequency between TABE-UNG and TABE at HEK4 and EMX1-site1 in HEK293T cells. (B) Comparison of editing frequency at indicated cytosines and adenines across TABE, ATBE and TABE-R33A. (C) Comparison of concurrent C-to-G and A-to-G editing across TABE, ATBE and TABE-R33A at EMX1-site1 and HEK4. (D) Comparison of editing frequency at indicated cytosines and adenines across GGBE1.0-GGBE1.5. (E) Comparison of concurrent C-to-G and A-to-G editing across GGBE1.0-GGBE1.5 at EMX1-site1 and HEK4. (F) Comparison of editing proportion of C-to-A/T/G across GGBE variants and GBE in HEK293T cells. Mean ± SEM (A-F) of all individual values of sets of n = 3 independent replicates are shown. Source data are provided as a Source Data file.

Supplementary figures

Supplementary Fig. 4

A

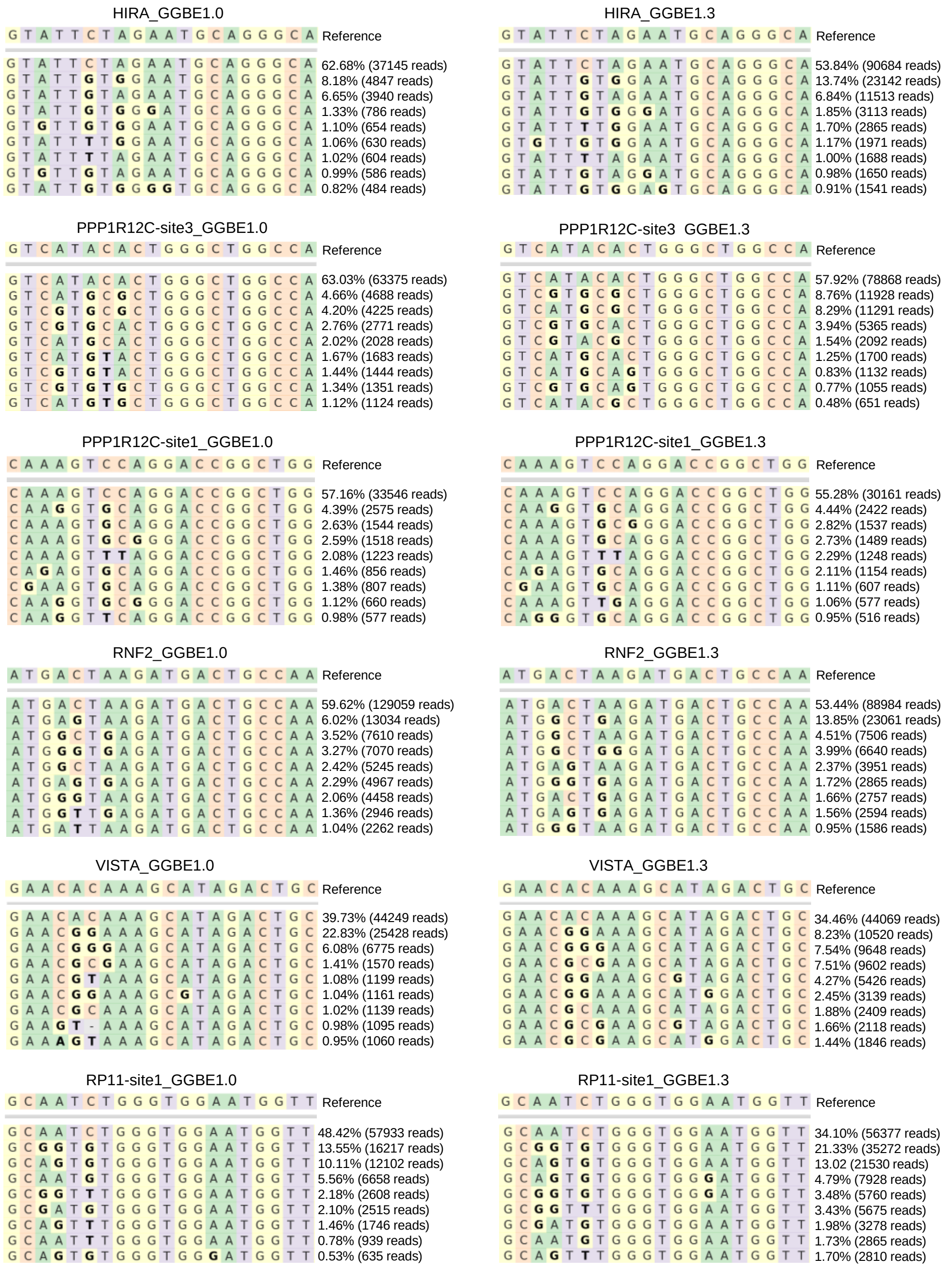

Supplementary figures

Supplementary Fig. 4

A

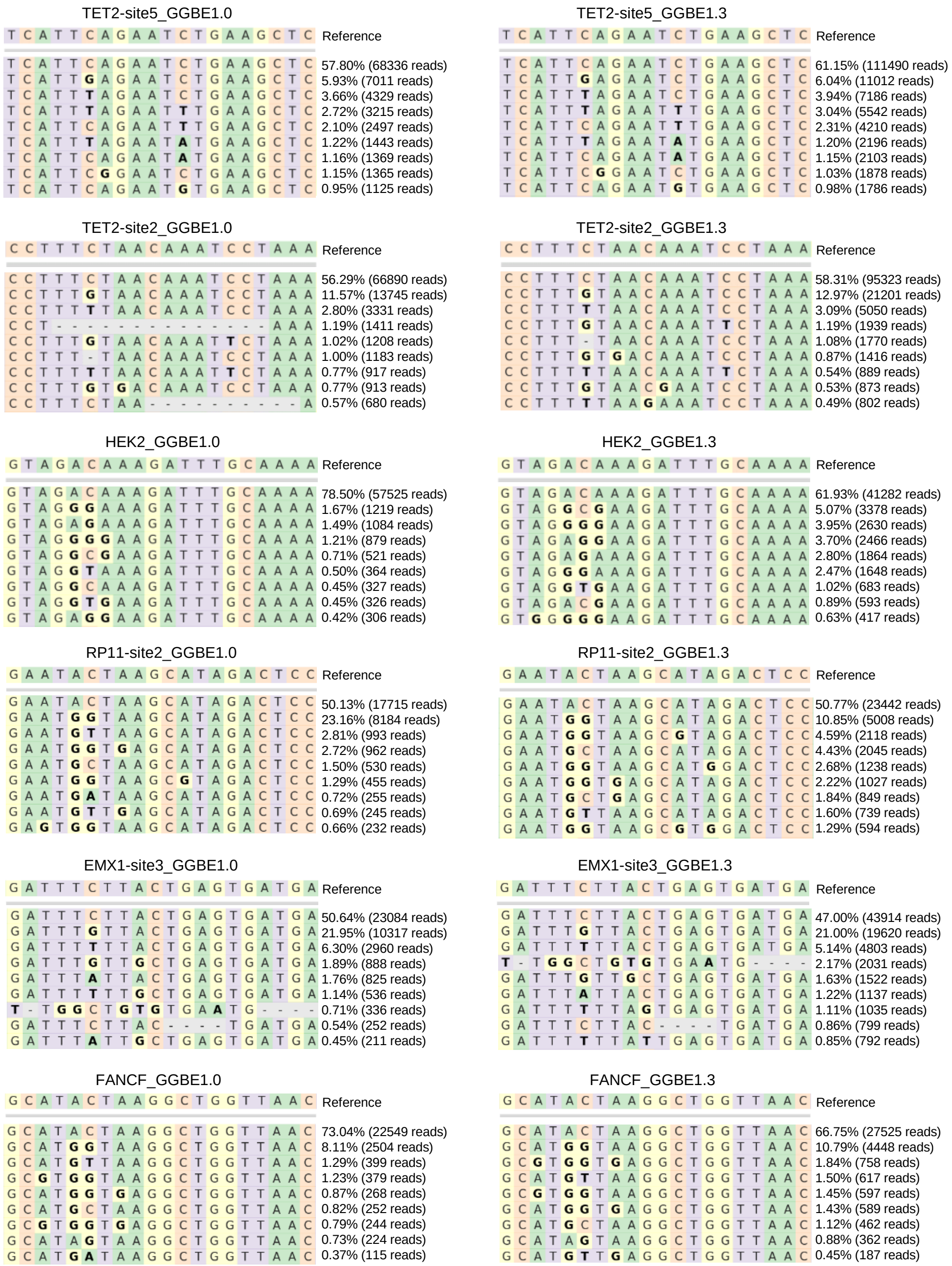

Supplementary figures

Supplementary Fig. 4

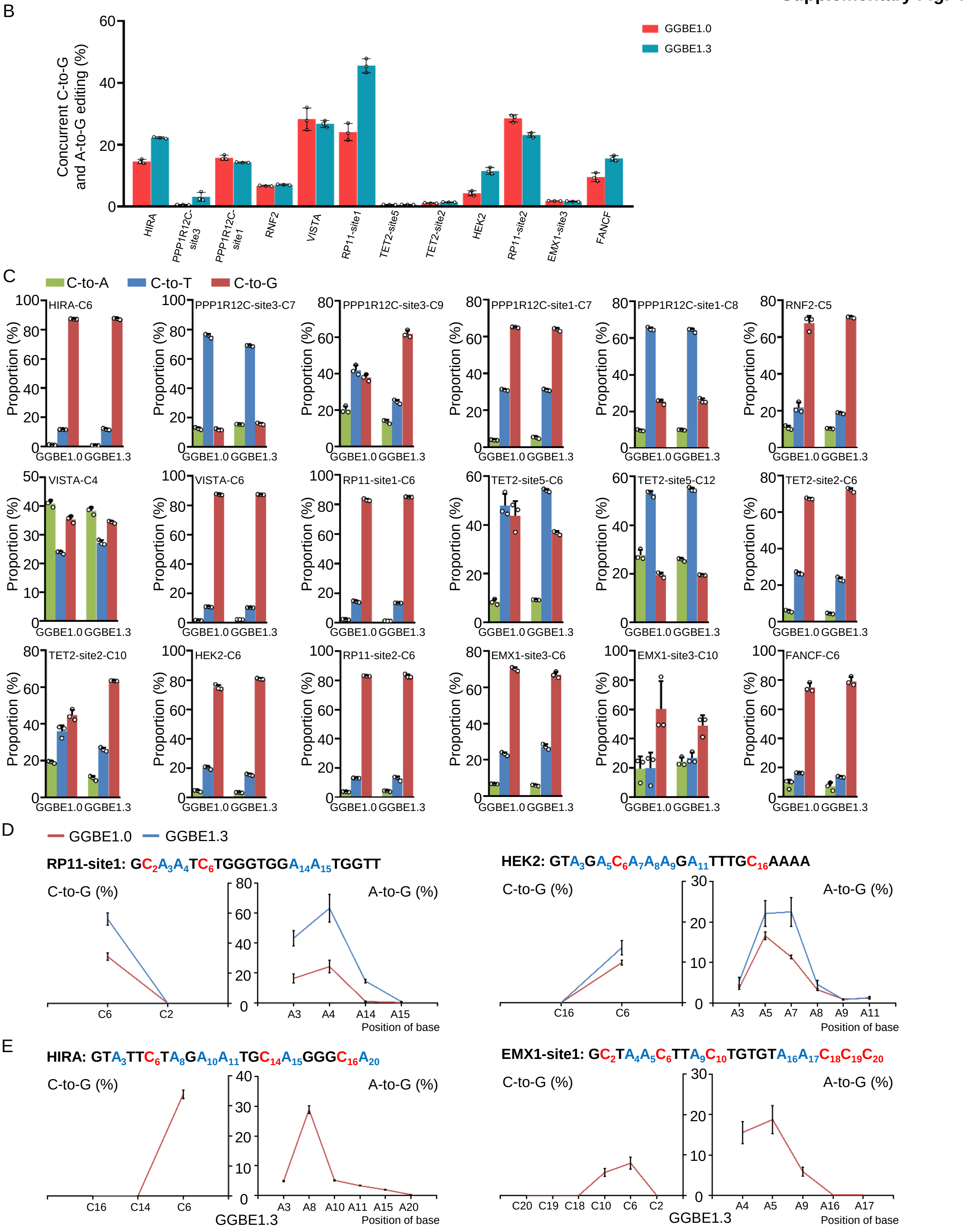

Supplementary figures

Figure S4. Further characterization of GGBE1.0 and GGBE1.3 in HKE293T cells. (A) The genotypes and reads and editing frequency of indicated genotypes in GGBE1.0 and GGBE1.3 across twelve genomic loci in HEK293T cells. (B) Comparison of concurrent C-to-G and A-to-G editing between GGBE1.0 and GGBE1.3 across twelve genomic loci. (C) Comparison of editing proportion of C-to-A/T/G between GGBE1.0 and GGBE1.3 across twelve genomic loci in HEK293T cells. (D) Comparison of editing efficiency between GGBE1.0 and GGBE1.3 in HeLa cells. (E) Editing frequency of GGBE1.3 at HIRA and EMX1-site1 in primary prostate carcinoma cells. Mean ± SEM (B-E) of all individual values of sets of n = 3 independent replicates are shown. Source data are provided as a Source Data file.

Supplementary figures

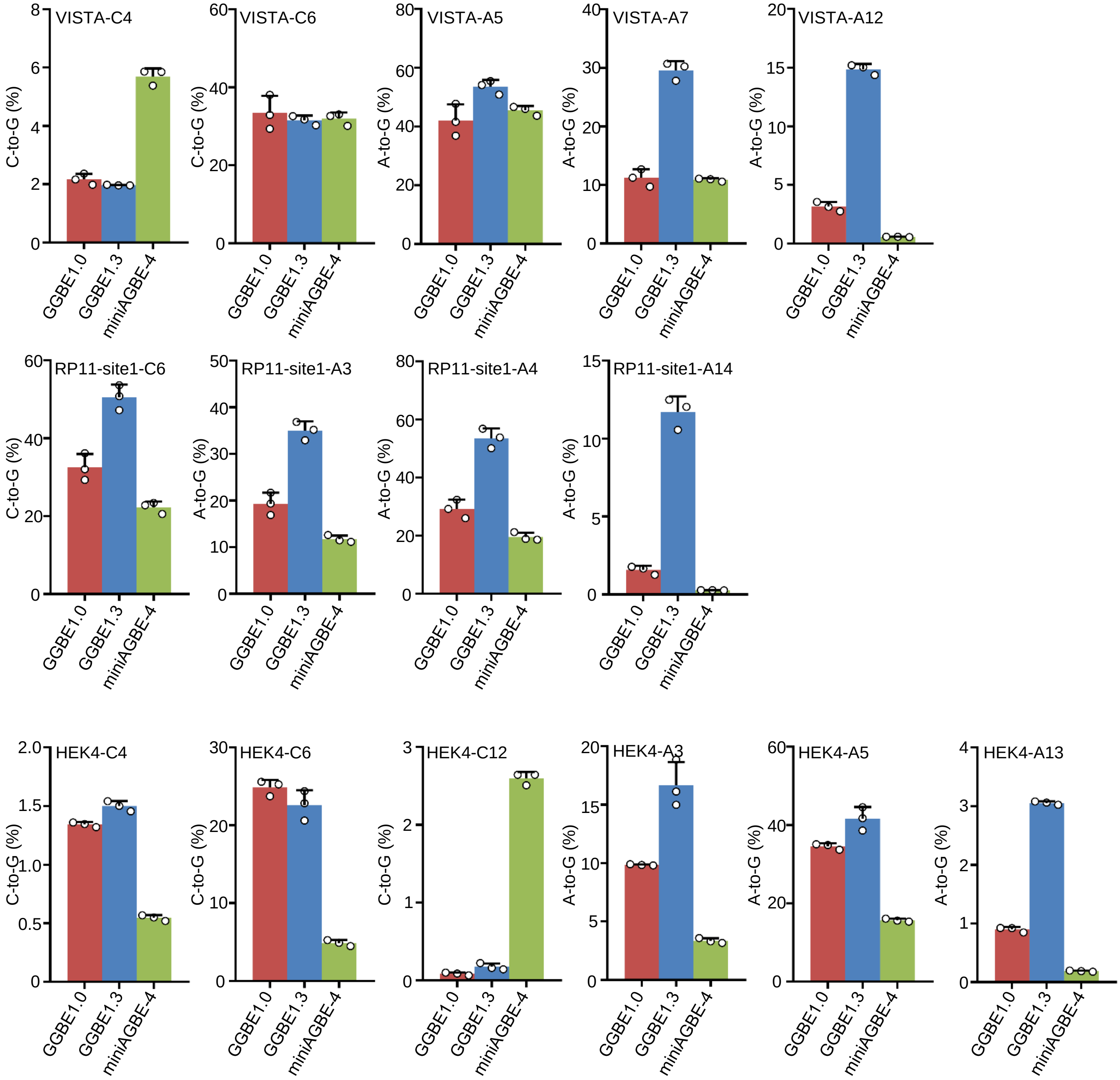

Figure S5. Comparison of editing efficiency at indicated cytosines and adenines across GGBE1.0, GGBE1.3 and minAGBE-4. Mean ± SEM of all individual values of sets of n = 3 independent replicates are shown. Source data are provided as a Source Data file.

Supplementary Fig. 6

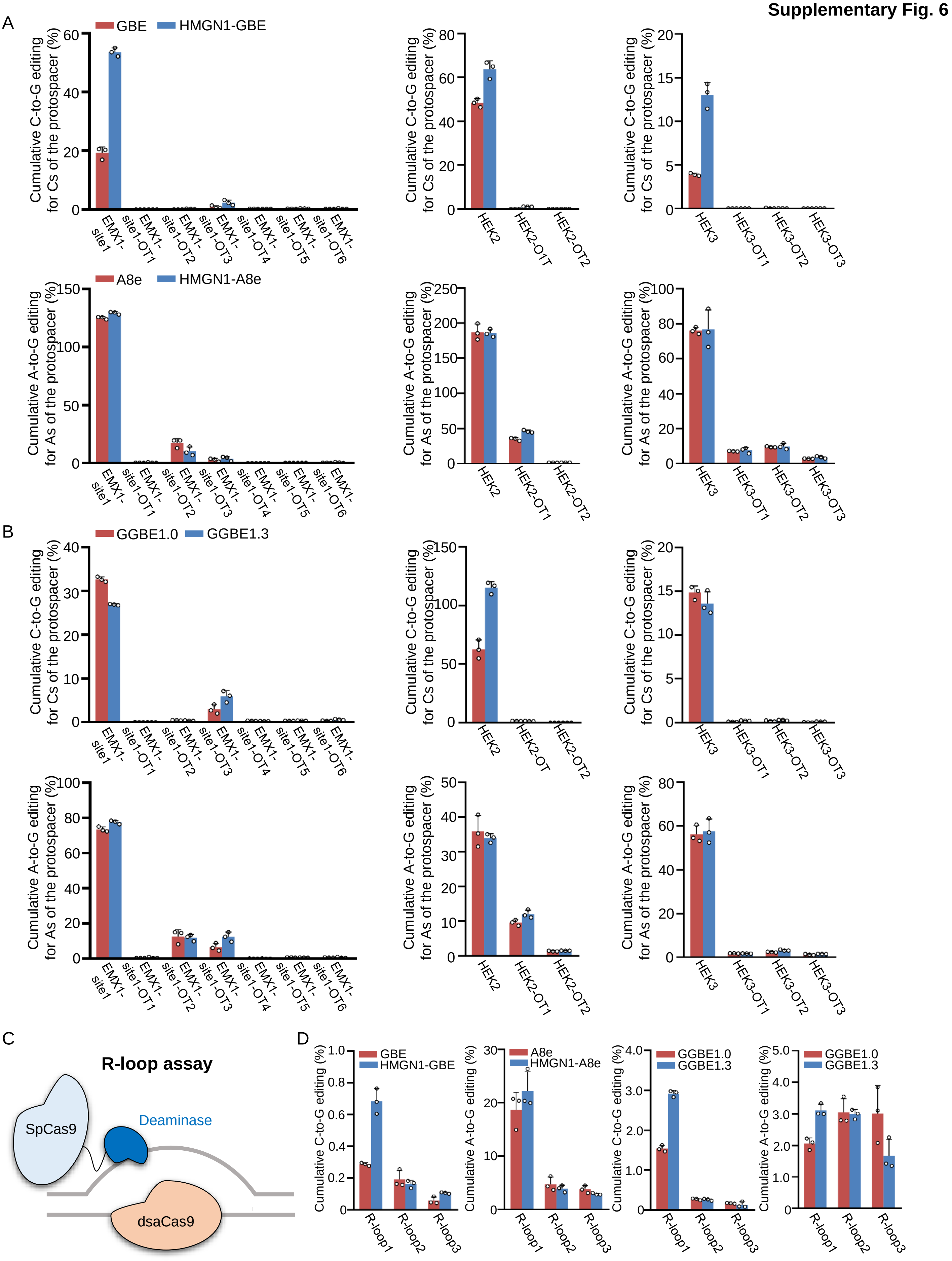

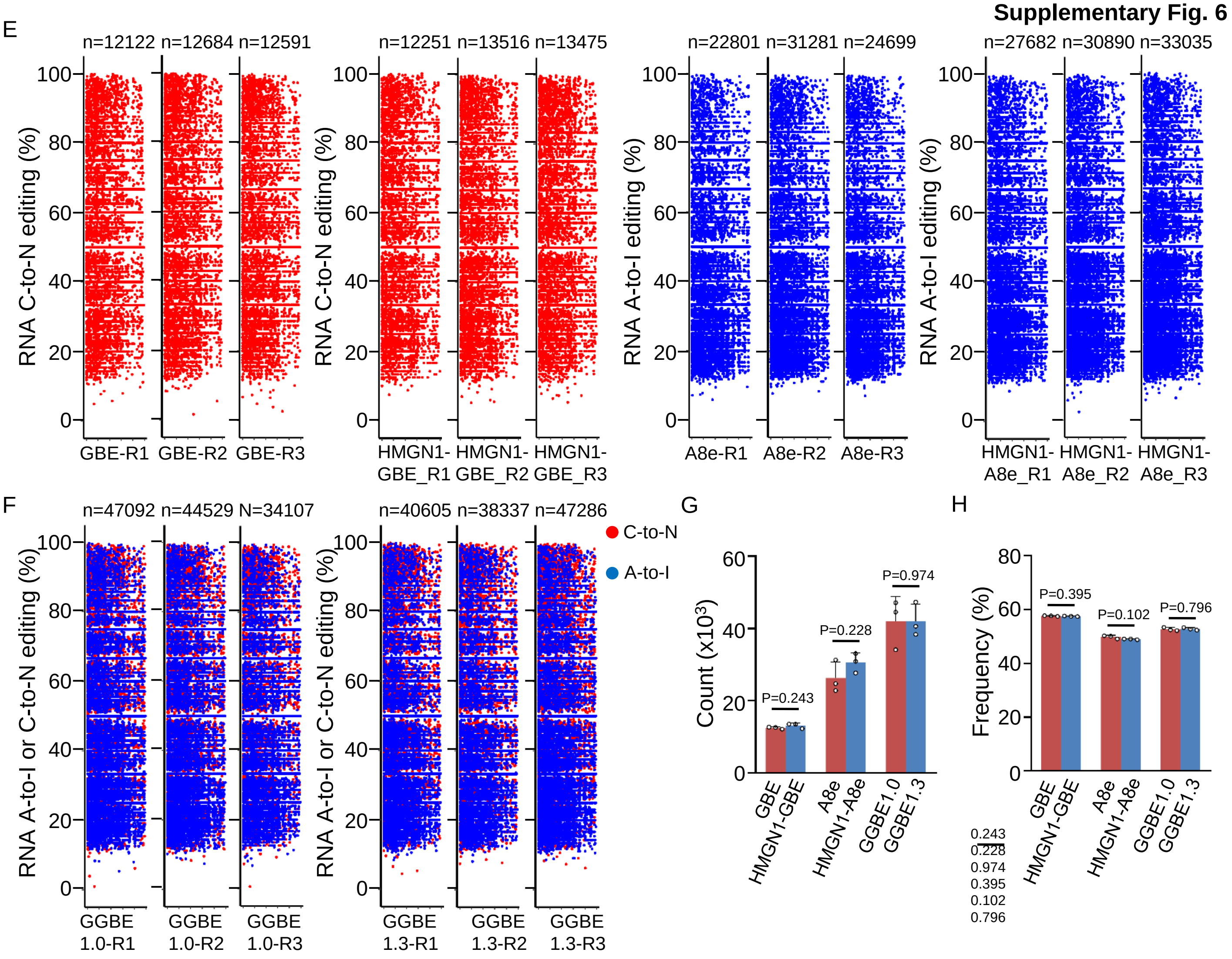

Figure S6. Off-target analysis of HMGN1 fused GBE, ABE and GGBE in HEK293T cells. (A) Cumulative C-to-G and A-to-G editing for Cs and As of the protospacer between GBE and ABE variants in HEK293T cells. (B) Cumulative C-to-G and A-to-G editing for Cs and As of the protospacer between GGBE1.0 and GGBE1.3 in HEK293T cells. (C) Schematic of R-loop assay for detection of Cas9-independent DNA off-target effects. (D) Cumulative C-to-G and A-to-G editing for Cs and As of the protospacer at three R-loops across GBE, A8e and GGBE variants. (E) Jitter plots from RNA-sequencing experiments in HEK293T cells showing efficiency of C-to-N or A-to-I conversions of GBE and A8e variants. Total number of modified bases is listed at the top. (F) Jitter plots from RNA-sequencing experiments in HEK293T cells showing efficiency of C-to-N or A-to-I conversions of GGBE1.0 and GGBE1.3. Total number of modified bases is listed at the top. (G) Comparison of numbers in RNA editing across GBE, A8e and GGBE variants. (H) Comparison of RNA editing frequency across GBE, A8e and GGBE variants. Mean ± SEM (a, b, d, g, h) of all individual values of sets of n = 3 independent replicates are shown. All statistical analysis for samples were conducted using unpaired Student's t-test (two-tailed) in GraphPad Prism 8. Source data are provided as a Source Data file.

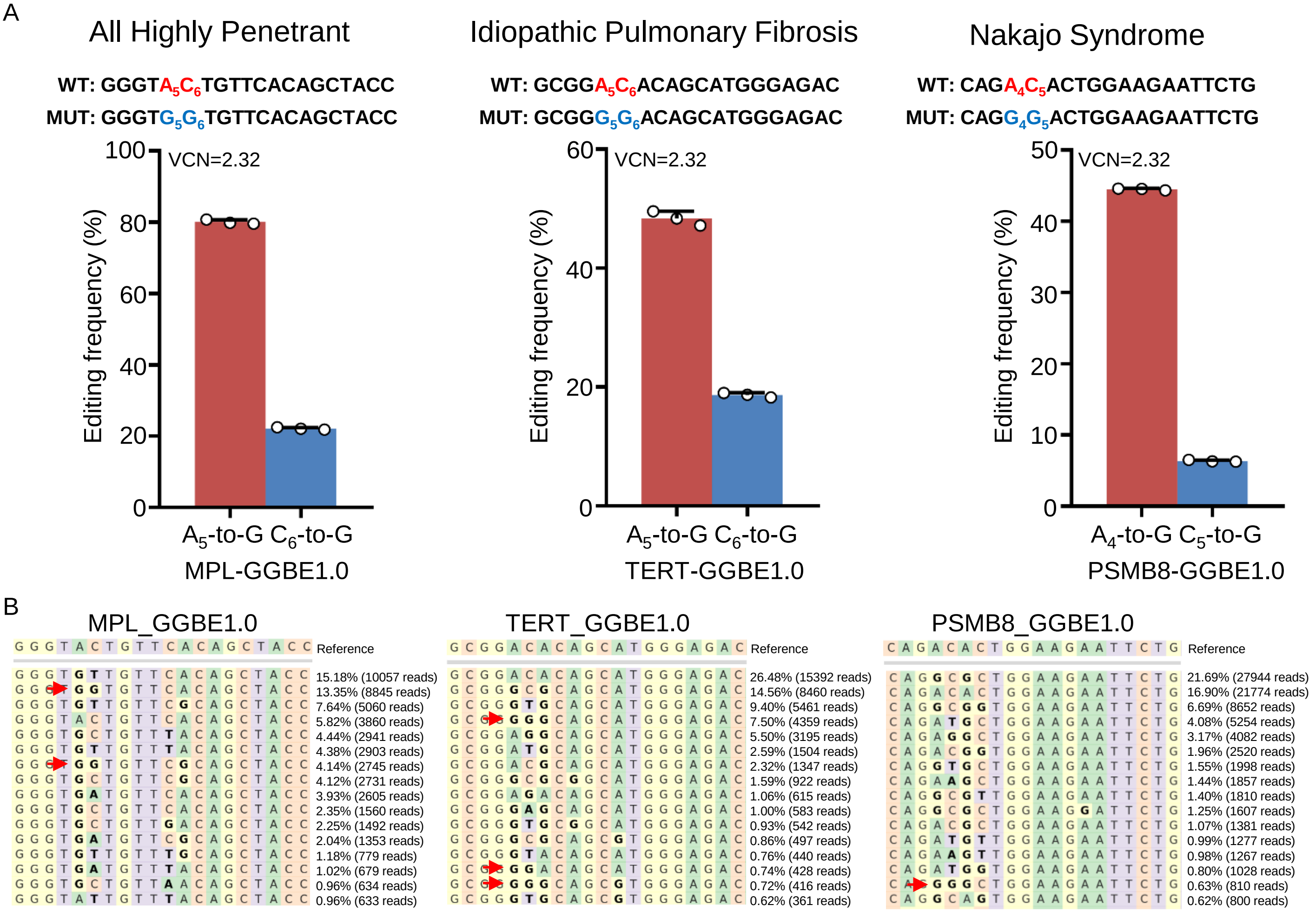

Figure S7. Potential application of GGBE in MNVs (A) A-to-G and C-to-G conversion by GGBE1.0 for MPL, TERT and PSMB8 MNVs. VCN, vector copy number. (B) The genotypes and reads and editing frequency of indicated genotypes at MPL, TERT and PSMB8 by GGBE1.0. Mean ± SEM (A) of all individual values of sets of n = 3 independent replicates are shown. Source data are provided as a Source Data file.
