# Supplementary material for: HMGN1 enhances CRISPR-directed dual-function A-to-G and C-to-G base editing
Source: Nat Commun. 2023 Apr 27;14:2430. doi: 10.1038/s41467-023-38193-2 (PMC10140177; doi:10.1038/s41467-023-38193-2)
Supplement: Supplementary file 2 — Description of Additional Supplementary Files [file 41467_2023_38193_MOESM2_ESM.pdf]

Title: Supplementary Data 1.
Description: Clonging PCR oligos.

Title: Supplementary Data 2.
Description:
Supplementary Data 2-1: Deep sequencing olgios of sgRNA.
Supplementary Data 2-2: Deep sequencing olgios of sgRNA On-target and Off-target site.

Title: Supplementary Data 3.
Description: Screened genes of GGBE targeted MNVs.
