# Supplementary material for: HMGN1 enhances CRISPR-directed dual-function A-to-G and C-to-G base editing
Source: Nat Commun. 2023 Apr 27;14:2430. doi: 10.1038/s41467-023-38193-2 (PMC10140177; doi:10.1038/s41467-023-38193-2)
Supplement: Supplementary file 3 — Reporting Summary [file 41467_2023_38193_MOESM3_ESM.pdf]

nature portfolio

Corresponding author(s): Changhao Bi

Last updated by author(s): Apr 15, 2023

Reporting Summary

Nature Portfolio wishes to improve the reproducibility of the work that we publish. This form provides structure for consistency and transparency in reporting. For further information on Nature Portfolio policies, see our Editorial Policies and the Editorial Policy Checklist.

Statistics

For all statistical analyses, confirm that the following items are present in the figure legend, table legend, main text, or Methods section.

n/a	Confirmed	
☐	☒	The exact sample size (*n*) for each experimental group/condition, given as a discrete number and unit of measurement
☐	☒	A statement on whether measurements were taken from distinct samples or whether the same sample was measured repeatedly
☐	☒	The statistical test(s) used AND whether they are one- or two-sided<br>*Only common tests should be described solely by name; describe more complex techniques in the Methods section.*
☒	☐	A description of all covariates tested
☒	☐	A description of any assumptions or corrections, such as tests of normality and adjustment for multiple comparisons
☐	☒	A full description of the statistical parameters including central tendency (e.g. means) or other basic estimates (e.g. regression coefficient) AND variation (e.g. standard deviation) or associated estimates of uncertainty (e.g. confidence intervals)
☐	☒	For null hypothesis testing, the test statistic (e.g. *F*, *t*, *r*) with confidence intervals, effect sizes, degrees of freedom and *P* value noted<br>*Give P values as exact values whenever suitable.*
☒	☐	For Bayesian analysis, information on the choice of priors and Markov chain Monte Carlo settings
☒	☐	For hierarchical and complex designs, identification of the appropriate level for tests and full reporting of outcomes
☒	☐	Estimates of effect sizes (e.g. Cohen's *d*, Pearson's *r*), indicating how they were calculated

Our web collection on statistics for biologists contains articles on many of the points above.

Software and code

Policy information about availability of computer code

Data collection	Deep sequence data was taken from Azenta
Data analysis	CRISPResso2 (v.2.0.45), STAR (v.2.7.10b), Picard (v.2.27.5) and GATK (v.4.3.0.0) are available on github (https://github.com/pinellolab/CRISPResso2; https://github.com/alexdobin/STAR; https://github.com/broadinstitute/picard; https://github.com/broadinstitute/gatk).

For manuscripts utilizing custom algorithms or software that are central to the research but not yet described in published literature, software must be made available to editors and reviewers. We strongly encourage code deposition in a community repository (e.g. GitHub). See the Nature Portfolio guidelines for submitting code & software for further information.

Data

Policy information about availability of data

All manuscripts must include a data availability statement. This statement should provide the following information, where applicable:

- Accession codes, unique identifiers, or web links for publicly available datasets
- A description of any restrictions on data availability
- For clinical datasets or third party data, please ensure that the statement adheres to our policy

HTS data have been deposited in the NCBI Sequence Read Archive database under accession codes PRJNA946328 (https://www.ncbi.nlm.nih.gov/bioproject/PRJNA946328). Source data are provided with this paper.

Human research participants

Policy information about studies involving human research participants and Sex and Gender in Research.

Reporting on sex and gender	Participant was determined based on self-report and the primary tissues was obtained from only one man diagnosed with prostate cancer, thus this section was not considered in our study
Population characteristics	The resected tissues was obtained with a man diagnosed with prostate cancer.
Recruitment	One patient that was diagnosed with prostate cancer was considered in our study.
Ethics oversight	Ethics Committee of Union Hospital, Tongji Medical College, Huazhong University of Science and Technology

Note that full information on the approval of the study protocol must also be provided in the manuscript.

Field-specific reporting

Please select the one below that is the best fit for your research. If you are not sure, read the appropriate sections before making your selection.

[x] Life sciences [ ] Behavioural & social sciences [ ] Ecological, evolutionary & environmental sciences

For a reference copy of the document with all sections, see nature.com/documents/nr-reporting-summary-flat.pdf

Life sciences study design

All studies must disclose on these points even when the disclosure is negative.

Sample size	All data are presented as means ± s.d. and analyzed with statistical method from three independent experiments.
Data exclusions	No data was excluded from data analysis, and all data obtained was used in the statistic analysis.
Replication	Data was obtained in the fashion of three independent biological replicates. All attempts for replication were successful.
Randomization	In the study, data from all performed experiments were collected and analyzed
Blinding	In the study, data from all performed experiments were collected and analyzed

Reporting for specific materials, systems and methods

We require information from authors about some types of materials, experimental systems and methods used in many studies. Here, indicate whether each material, system or method listed is relevant to your study. If you are not sure if a list item applies to your research, read the appropriate section before selecting a response.

Materials & experimental systems

n/a	Involved in the study	
[ ]	[x]	Antibodies
[ ]	[x]	Eukaryotic cell lines
[x]	[ ]	Palaeontology and archaeology
[x]	[ ]	Animals and other organisms
[x]	[ ]	Clinical data
[x]	[ ]	Dual use research of concern

Methods

n/a	Involved in the study	
[x]	[ ]	ChIP-seq
[x]	[ ]	Flow cytometry
[x]	[ ]	MRI-based neuroimaging

Antibodies

Antibodies used	Cas9, dilution: 1:2000, Cat:AF0123, Lot:071822221109, Beyotime Biotechnology; GAPDH, dilution: 1:10000, Cat:AC002, Lot:3500033004, Abclonal; Secondary antibody, HRP-conjugated Affinipure Goat Anti-Mouse IgG (H+L), dilution: 1:5000, Cat:AS003, Lot:930003001, ABclonal.
Validation	Cas9: IF (human, HEK293T), WB (human, HKE293T);GAPDH: IF (Mouse, NIH3T3), IHC (human kidney; mouse kdiney), WB (human, Hela, HEK293T, HepG2;Mouse liver and spleen; Rat liver and kidney); HRP-conjugated Affinipure Goat Anti-Mouse IgG (H+L): WB (human and mouse cell lines, HeLa, HEK293T, HepG2, Jurkat, K562, HSC-T6, PC-12, NIH3T3, 4T1).

Eukaryotic cell lines

Policy information about cell lines and Sex and Gender in Research

Cell line source(s)	HEK293T from ATCC
Authentication	No cell lines were authenticated
Mycoplasma contamination	Cells were not tested for mycoplasma contamination.
Commonly misidentified lines (See ICLAC register)	No cell lines were used that are in the ICLAC register.
